# Organic seed priming with curtailed seed rate compensated wheat grains productivity by upgrading anti-oxidant status against terminal drought at flowering and milking

**DOI:** 10.1038/s41598-024-54767-6

**Published:** 2024-02-28

**Authors:** Hamid Nawaz, Haseeb-ur Rehman, Muhammad Zahid Ihsan, Muhammad Shahid Rizwan, Nazim Hussain, Basharat Ali, Rashid Iqbal, Muhammad Usama Hasnain, Mohamed S. Elshikh, Jawaher Alkahtani, Muhammad Arslan

**Affiliations:** 1 Cholistan Institute of Desert Studies, The Islamia University of Bahawalpur, Bahawalpur, 63100 Pakistan; 2 Department of Agronomy, Bahauddin Zakariya University, Multan, 60800 Pakistan; 3 Agronomic Research Station, Bahawalpur, 63100 Pakistan; 4 Department of Agronomy, The Islamia University of Bahawalpur, Bahawalpur, 63100 Pakistan; 5 Institute of Plant Breeding and Biotechnology, MNS-University of Agriculture, Multan, 66000 Pakistan; 6 Department of Botany and Microbiology, College of Science, King Saud University, 11451 Riyadh, Saudi Arabia; 7 Institute of Crop Science and Resource Conservation (INRES), University of Bonn, 53113 Bonn, Germany

**Keywords:** Wheat, MLE30-priming, Curtailed seed rate, Anti-oxidant, Grain’s yield, BCR, Abiotic, Drought

## Abstract

Terminal irrigation drought stress is one of the most drastic abiotic stress to diminish the wheat crop development and grains yield in arid regions of the world. The use of moringa leaf extract (MLE30) via seed priming technique is investigated as an organic and sustainable approach for the mitigation of drought stress along with curtailed seed rate in wheat crop. The study investigated the interaction of organic seed priming: control (dry seeds), hydro-priming, MLE30-priming, seed rate: recommended @ 125 kg ha^−1^, curtailed @ 25 kg ha^−1^, and terminal irrigation drought (TID): normal irrigation, mild-TID, severe-TID in wheat crop at agronomic research station, Bahawalpur, Pakistan during the wheat winter season of 2021–2022 and 2022–2023. The application of organic MLE30-priming with curtailed seed rate enhanced antioxidant enzyme activity especially total soluble proteins by 15%, superoxide dismutase by 68%, peroxidase by 16%, catalase by 70%, ascorbic acid by 17% and total protein contents by 91% under severe-TID. Yield and yield-related morphological attributes performed better in MLE30-priming as compared to hydro-priming. An effective trend was observed in the plant's chlorophyll contents, K^+^, and water use efficiency after being treated with MLE30-priming followed by hydro-priming under curtailed seed rate. The higher benefit–cost ratio and net income return were observed with the application of MLE30-priming with curtailed seed rate under mild-TID and severe-TID. So, it is suggested to adopt the MLE30-priming technique along with a curtailed seed rate for improving the crop establishment, stress regulation, and economic return under limited availability of irrigation water. The project findings recommended that the application of exogenous application of organic MLE30-seed priming favored and compensated the maximum wheat grains production under curtailed seed rate @ 25 kg ha^−1^ and induced terminal drought stress at flowering and milking conditions.

## Introduction

Water scarcity is one of the major limiting factors for crop production and becomes a serious threat to food security^1^. Water stress was assumed to be a catalyst of severe starvation in history. The food demand was gradually increasing per capita due to the sudden shortage of usable water availability. The impact of drought severeness is categorized by the rate and interval of rainfall, soil evaporation losses, and lack of water harvesting approaches^2^. Soil moisture stress disrupted and induced the alteration in the various plant biochemical, physiological, and morphological processes including cell divisions, cell elongations, turgor pressure, photosynthetic assimilates translocation, anti-oxidant behavior, nutrient uptake, and grains settings^3^. The intensity of drought can be observed in all the phenological growth stages of wheat crop, but the terminal drought stress at reproductive phases impaired the grain's productivity^4^. Terminal irrigation drought (TID) may be classified into mild-TID (at the flowering stage) and severe TID (at flowering and milking stages) during the wheat field crop production^5^.

The phenomenon of plant drought tolerance is the ability to survive by using various adaptations, mechanisms, and sustainable smart approaches under a water-stress environment^6^. In general, plants have altered their lifestyles through adaptive processes resulting in changes in appearance including escape, flexibility, and avoidance^7^. Plants adjusted their physiological behavior by improving osmotic pressure, and stability in cell membranes^8^. The tolerant plants also maintained internal biochemical production in the form of proline, auxins, ethylene, activated stress proteins, transcript values, and metabolism at the molecular level^9^. In addition, drought stress can be mitigated by using the various sustainable agronomic smart approaches as exogenous applications of growth enhancers, osmoprotectants, mineral plant nutrients, curtailed seed rate, water use efficient cultivars, appropriate cropping patterns, and organic & synthetic mulches^10^. However, all these practices are costly, technical, and quite difficult for the farmer’s community to manage the drought stress in field conditions^11^.

The use of the seed priming approach is one of the cost-effective and sustainable smart agronomic techniques to mitigate the drastic impact of plant grain development phases under terminal irrigation drought stress^12^. The treated primed seeds can upgrade the plant tolerance via germination potential, vigorous seedling establishments, and ameliorate the anti-oxidant defense system to protect the cellular oxidative damages^13^. Seed priming is the procedure of soaking the seeds depending on the low water potential solution and duration^14^. Different seed priming methods have been explored based on the utilization of priming agents and can be classified as hydro-priming, osmo-priming, matri-priming, osmo-hardening, hardening, hormonal priming, etc^15^. The applied seed priming treatments with naturally occurring plant growth enhancers known as “organic seed priming” is the most economical, practical, and productive strategy for augmenting the stress tolerance^1^. Moringa leaf extract (MLE30) is the newly emerging priming agent termed as organic MLE30-priming technique having rich contents of Zeatin (Cytokinin) useful for mitigating the production of reactive oxygen species (ROS) during the terminal irrigation drought condition^16^.

Various scientists and field researchers diagnosed that the optimum wheat crop establishment plays a significant role in obtaining the economical and profitable grain yield^17^. The applied seed rate is the main determining aspect for capturing the crop input resources and is important, especially for wheat production under the farmer’s control^18^. The seed rate is directly proportional to the plant density, strongly related to the regional climate, soil condition, sowing methodology, and time. The use of a curtailed seed rate favored in producing the maximum fertile tillers leads to healthy spike development, grain weight, and final grain yield^7^. Newly developed hybrid wheat may require lower seeding rates than traditional cultivars^19^. Therefore, the two years of field project study were planned to investigate the interactive potential effect of various seed priming techniques including organic MLE30-priming along with curtailed seed rate in enhancing the wheat grains productivity under the induced terminal irrigation drought stress conditions.

## Materials and methods

### Preparation for organic MLE30-priming

Fresh moringa leaves were collected from mature trees after noon at the Agronomic Research Area, in the Department of Agronomy, Bahauddin Zakariya University, Multan, Pakistan. The collected leaves were cleaned and washed with distilled water. Moringa leaves were frozen in the laboratory chiller refrigerator at – 80 °C and pressed through the locally fabricated manual machine to get the extract. Then, the extract was centrifuged by using the standard 8000 rpm for 20 min and diluted with distilled water 30 times after purifying. Wheat seeds were soaked for seed priming processing; in the hydro-priming technique, measured seeds as per applied treatments and plot size were dipped in aerated water. For organic MLE30-priming; seeds were soaked with mixing MLE30 solution by maintaining the ratio 1:30 (MLE30: water) respectively. An electric aquarium vacuum pump was used for aeration during the seed priming procedure for 8 hours^17^.

### Soil physico-chemical analysis and meteorological data

**Figure 1:**
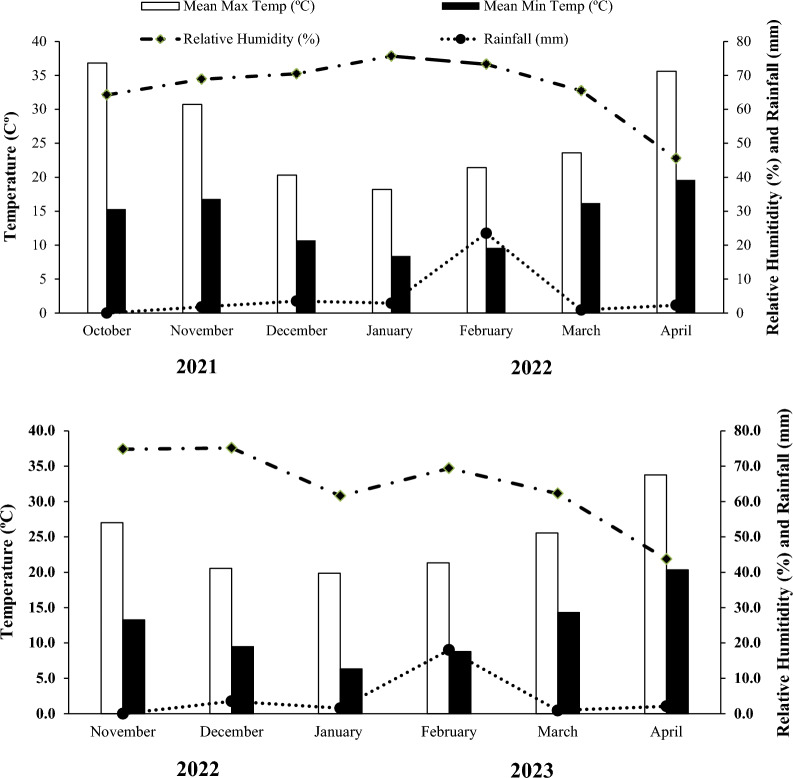
Meteorological data during wheat phenological growing seasons of 2021–2022 and 2022–2023. Metrological department, Cholistan Institute of Desert Studies (CIDS), Pakistan.

Different three locations were selected at the experimental site to collect the samples by using the stainless-steel spade and preserved in the polythene envelopes. The standard procedure was described for measuring the soil characteristics and physio-chemical properties of collected field soil samples^16^. After analysis, observations were noted and presented in Table 1. The climate of Multan is an arid and sub-tropical region having the description of 71.53°E, 30.02°N, and 123 m above sea level. Weather data is illustrated in the average rainfall, relative humanity, maximum and minimum temperature during the phenological growth period of the wheat crop during both years 2021–2022 and 2022–2023. Figure 1 describes the minor rainfall observed during the crop growth and development stages, but its intensity was negligible during the applied terminal irrigation drought stress conditions.

**Table 1:** Average soil characteristics & phyico-chemical properties during the growing season 2021–2022 & 2022–2023.

Soil analysis components	Units	Values
Soil belongs to “Lyallpur” soil series having following features given as below		
Sand	%	41.9
Silt	%	60.01
Clay	%	34.02
Texture	–	Sandy clay loam
Organic matter	%	0.64
Saturation	%	36.10
Nitrogen	%	0.05–0.09
Phosphorus	mg kg^−1^	1.8
Potassium	mg kg^−1^	5.24
Zinc	mg kg^−1^	0.35–0.40
EC	dS m^−1^	0.66
pH	–	8.12

Laboratory tests were performed in the Soil Science Section, RARI, Bahawalpur, Pakistan.

### Experimental design and crop husbandry

Field experiments were conducted at the Agronomic Research Station, Bahawalpur, Pakistan, Pakistan during the winter season 2021–2022 & 2022–2023 of wheat crop. The trials were carried out by using a randomized complete block design (RCBD) with factorial arrangements and replicated thrice. The plot size and plant-plant distance were recommended having dimensions 3 m × 5 m and 21.5 cm respectively. The seeds of the Ghazi-2019 wheat cultivar were used as a test species having the line-line distance of 23 cm.

This project study consisted of the followings factors as F_A_: seed priming i.e., control (dry seeds), hydro-priming, MLE30-priming F_B_: seed rate i.e., recommended @ 125 kg ha^−1^, curtailed @ 25 kg ha^−1^ and F_C_: terminal irrigation drought (TID) i.e., normal irrigation condition at tillering (T), booting (B), flowering (F), milking (M), mild-TID {F}, sever-TID {F + M}. The experimental field was prepared after the application of water irrigation at a depth 10 cm and attained the workable condition for sowing wheat crop. Then, the seedbed was arranged and ploughed twice followed by planking. A digital tensio-meter was used to maintain the drought stress condition after each application of water irrigation at wheat critical growth stages. Trials sowing time was the first fortnight of November and harvesting time was the second fortnight of April during both years year-I & II. All the agronomic inter-cultural and plant protection measures were practiced as per requirement. A square meter was used to determine the average number of fertile/productive tillers, then grains spike^−1^ was calculated from selected healthy twenty-five spikes, and the weight of 1000-grains was recorded. After harvesting, the harvested wheat material was cut by hand in each experimental unit, tightly tied in bundles, and dried in the sun for 7 days. Each of the bundles was weighed for biological yield and then threshed for grain yield.

### Biochemical analysis

To determine the biochemical analysis, healthy flag leaves were selected and then collected shortly after 7–10 days of the last water irrigation at a morning temperature of 20 ± 5 °C and stored in polyethylene bags at − 80 °C in the refrigerator for antioxidant analysis. The modified protocol described by^20^ was used to measure the total soluble proteins (TSP). For this, fresh plant leaves material of 0.5 g was grounded by mixing the prepared 1 mL extraction buffer (pH 7.2) in pastor mortar (pre-chilled). The required cocktail protease inhibitor (1 µM) was mixed in the buffer solution before extraction of proteins. The stock solution of phosphate buffer saline (PBS) was prepared with adding 10 mM Na_2_HPO_4_ + 2 mM KH_2_PO_4_ + 2.7 mM KCl + 1.37 mM NaCl up-to 1 L. HCl was gradually added for adjusting the PBS pH 7.2. After this, sample of ground leaf material was run at digital centrifuged machine at 12,000×*g* for 5 min and removed the debris material from centrifuge tube. The remaining supernatant sample material was separately preserved at laboratory temperature for measuring the soluble proteins. The standard curve was drafted at 10, 20, 30, 40 and 50 µg mL^−1^ from bovine serum albumin (BSA) after mixing the 400 µL Dye stock followed by deionized distilled water. The given sample was carefully mixed at vortex machine and incubating at laboratory room temperature for 30 min. Nano spectrophotometer was used to observe TSP by adjusting the absorbance at 595 nm.

Peroxidase (POD) and catalase (CAT) contents was measured by preparing the reaction mixture with the addition of 400 µL guaiacol (20 mM), followed by 500 µL H_2_O_2_ (40 mM), 2 mL phosphate (50 mM) in 100 µL sample extract. The absorbance values for POD contents were measured at 470 nm for 20 s up-to 5 mins^21^. Similarly, CAT contents were observed after decomposition of H2O2 in reactive mixture followed by absorbance at 240 nm (30 s for 5 min)^22^.

Superoxide dismutase (SOD) was determined after preparing the reaction mixture having 50 µL samples extract followed by mixing 1mL nitroblue tetrazolium (NBT) (50 µM), 500 µL methionine (13 mM), 1mL riboflavin (1.3 µM), 950 µL (50 mM) phosphate buffer and 500 µL EDTA (75mM). The samples were kept for reaction at 30 W fluorescent lamp illuminations and switch on the fluorescent lamp for 5 min. The blue formazane was appeared after NBT photo reduction and then SOD contents were observed at 560 nm absorbance^22^.

Ascorbic acid (AsA) was calculated as given supernatant sample material (200 µL) was prepared with the addition of 1.4 mL PBS and incubated for 1 min at laboratory temperature. After this, reagents (0.4 mL trichloroacetic acid (10%), 44% phosphoric acid (0.4 mL), 0.2 mL 2, 2-biphenyl and 0.2 mL FeCl3 (3%)) were mixed well and incubated again for 60 min at 35 °C. And, the absorbance of sample reactive mixture for AsA was noted at 525 nm^23^.

Gallic acid was used as a standard solution for determining the total phenolics contents. Sample was homogenized with 5 mL acetone (80%). The prepared sample was filtered followed by extraction with acetone up-to 10 mL. After this, the standard sample (20 µL) was mixed with deionized distilled water (1.58 mL), Folin-Ciocalteu reagents (100 µL) within 30 s up-to 8 min and after adding sodium carbonate (300 µL), the reactive mixture was left for 30 min at 40 °C. TPC was measured at 760 nm absorbance^24^.

Moreover, the given standard procedures were used to measure the leaf chlorophyll contents (“*a*” and “*b*”)^25^, potassium (K^+^) contents^26^, and water use efficiency (WUE)^27^.

### Benefit–cost ratio (BCR)

An economic analysis was executed after the application of the organic MLE30-priming approach along with curtailed seed rate in enhancing the wheat grains yields under terminal irrigation drought. It contained total expenditures of wheat production including field land rent, prepared workable seedbed, seed rate, sowing rate, fertilizer rate, water irrigation charges, protection measured charges, and crop harvesting charges. Gross income was calculated by applying the country market price of wheat grains and straw. Net income and benefit–cost ratio were measured by using the following formulas^1^ described as;

$${\text{Net \, income }} = {\text{ Gross \, income }}{-}{\text{ Total \, expenditures}}$$

$${\text{BCR }} = {\text{ Gross \, income }}/{\text{ Total \, expenditures}}$$

### Principal component analysis (PCA)

Biplot analysis for observed parameters was described by following^28^. This technique involves dividing the correlation coefficients between direct and indirect effects by alternative means for random variables on the resultant variables.

### Statistical analysis

The procedure described to analyze the data under Fisher’s analysis of variance technique^29^. LSD was used to test and compared the treatment's means at 5% probability level via Statistix v8.1 software. MS excel office program 2019 was used for graphically representation.

### Ethical approval

The plant collection and use were under all the relevant guidelines.

## Results

ANOVA expressed the significant interaction of studied traits analyzed by using RCBD factorial design under *p* test after applied treatments having organic seed priming with curtailed seed rate under various terminal irrigation drought (TID) during the both years of trials shown in the Table 2.

**Table 2:** Three-way ANOVA table of studied traits analyzed by using RCBD factorial design under *p* test.

Source of variance	Degree of freedom	Productive tillers	Grain’s spike^−1^	1000 Grains weight	Grain’s yield	Biological yield	Harvest index	Total soluble protein	Superoxide dismutase	Peroxidase	Catalase	Ascorbic acid	Total phenolic contents	Chlorophyll *a*	Chlorophyll *b*	K^+^	Water use efficiency
Year-I (2021–2022)																	
Rep	2	–	–	–	–	–	–	–	–	–	–	–	–	–	–	–	–
Dr	2	4289**	1290.76**	149.12**	951.38**	3647.40**	20.88**	36.58**	251.05**	75.41**	12,544.5**	190.37**	122.26**	146.97**	141.81 **	1309.51**	193.00**
Pr	2	213**	299.09**	135.18**	303.40**	300.38**	0.45^NS^	86.62**	109.27**	238.30**	2223.08**	478.34**	12.75**	145.56**	168.24**	80.67**	193.47**
SR	1	5.82**	6539.14**	125.42**	768.36**	761.51**	15.18^NS^	35.31**	188.39**	271.17**	1277.06**	228.26**	9.67*	75.77**	86.13**	289.00**	108.64**
Dr*Pr	4	77**	217.64**	17.08**	83.24**	82.23**	1.87^NS^	2.55^NS^	132.73**	44.31**	1170.76**	20.82**	2.10^NS^	3.83*	42.77**	13.14**	9.70**
Dr*SR	2	0.97*	301.65**	26.89**	52.52**	52.22**	0.13NS	3.26^NS^	9.69**	17.87**	452.52**	7.03*	3.01^NS^	38.25**	8.62**	29.85**	4.23*
Pr*SR	2	58**	290.02**	0.70**	153.83**	151.76**	4.14*	9.87*	6.98*	41.32**	80.89**	15.73**	4.87*	5.28*	30.79**	1.32NS	23.65**
Dr*Pr*SR	4	23.8 **	341.33**	9.26**	60.55**	60.13**	1.78^NS^	1.17*	11.64*	7.33*	205.69**	52.57**	0.11*	4.53*	9.47**	10.20**	6.85**
Error	34	–	–	–	–	–	–	–	–	–	–	–	–	–	–	–	–
Total	53	–	–	–	–	–	–	–	–	–	–	–	–	–	–	–	–
CV	–	1.51	0.62	3.43	2.45	1.97	6.52	10.22	5.11	2.63	0.85	2.27	24.43	11.47	5.66	2.76	5.83
Year-II (2022–2023)																	
Rep	2	–	–	–	–	–	–	–	–	–	–	–	–	–	–	–	–
Dr	2	180.81**	77.27**	525.84**	306.07	593.57**	2.40^NS^	125.65**	639.98**	326.84**	9262.05**	69.83**	412.87**	17.14**	49.06**	202.32**	191.58**
Pr	2	2034.38**	11.62**	335.77**	163.38	209.46**	9.28*	7.37*	52.20**	1125.35**	9914.33**	140.54**	62.01**	26.34**	57.84**	9.50**	47.81**
SR	1	999.03**	415.13**	356.77**	89.90	125.38**	32.02**	41.84**	551.89**	352.80**	1300.59**	139.26**	98.47**	39.67**	59.07**	15.64**	16.64**
DR*Pr	4	57.60**	2.88**	110.96**	7.50	13.81**	5.99*	46.92**	54.13**	3.88NS	169.48**	26.78**	13.99**	23.90 **	20.47**	5.00**	5.68**
Dr*SR	2	381.52**	11.33**	128.29**	22.99	32.08**	0.56^NS^	17.30**	82.58**	38.10**	256.72**	30.21**	25.30**	2.99NS	3.54*	1.29 NS	5.98**
Pr*SR	2	64.65**	29.16**	63.10**	88.21	119.15**	9.75*	46.71**	1.63^NS^	162.12**	246.06**	10.02**	7.38*	2.24NS	0.21^NS^	1.37NS	1.79NS
Dr*Pr*SR	4	49.43**	25.22**	5.35*	10.93	7.65*	3.67^NS^	2.93*	28.44**	33.05**	44.38**	26.87**	1.24*	5.51**	8.76**	0.52*	2.64*
Error	34	–	–	–	–	–	–	–	–	–	–	–	–	–	–	–	–
Total	53	–	–	–	–	–	–	–	–	–	–	–	–	–	–	–	–
CV	–	1.71	2.46	2.17	6.54	3.93	4.44	2.04	3.71	1.26	0.99	2.58	6.23	11.10	9.05	15.81	6.27

*Significant @p ≤ 0.05, **Highly significant @p ≤ 0.01, *NS* non-significant @p ≤ 0.05, *Rep* Replication, *Dr* Drought, *Pr* Priming, *SR* Seed rate, *CV* Coefficient of variance.

### Yield and yield-related morphological attributes

Table 3 illustrated the significant interaction between the applied treatments seed priming with curtailed seed rate on morphological parameters under induced terminal irrigation drought (TID) stress. Results showed that wheat productive tillers were diminished under applied severe-TID and mild-TID as compared to normal irrigation conditions but the MLE30-priming technique significantly improved the productive tillers with curtailed seed rate (25 kg ha^−1^) during the year-II as per year-I presented in the Table 3. It was observed that plants treated MLE30-priming technique with curtailed seed rate obtained the highest number of grains spike^−1^, and 1000-grains weight after hydro-priming under normal irrigation conditions followed by mild-TID and severe-TID as compared to lowest in control during the second year of study shown in the Table 3. The observations exhibited that maximum grain yield was obtained under applied treatment MLE30-priming in the normal irrigation condition during the 1st year of trial as compared to the 2nd year followed by mild-TID and severe-TID with curtailed seed rate demonstrated in Table 3. Wheat plants treated with MLE30-priming technique demonstrated the greater biological yield as per hydro-priming under normal irrigation conditions as well as Mild-TID and severe-TID conditions and least in control treatment during the year-I after year-II of the project study. The interactive effect of seed priming techniques, seed rates, under terminal irrigation drought for harvest index (HI) was perceived as non-significant during both years of trials presented in Table 3.

**Table 3:** Evaluate the organic seed priming with curtailed seed rate on morphological yield attributes of wheat crop under terminal irrigation drought (TID).

Seed priming		Control		Hydro-priming		MLE30-priming			Mean
Seed rate/TID		Recommended 125 kg ha^−1^	Curtailed 25 kg ha^−1^	Recommended 125 kg ha^−1^	Curtailed 25 kg ha^−1^	Recommended 125 kg ha^−1^	Curtailed 25 kg ha^−1^		
Productive tillers (m^2^)									
Year-I	Normal	330.67d ± 1.75	309.00f ± 1.25	341.00c ± 0.27	350.67b ± 1.13	342.67c ± 0.68	360.00a ± 0.54		283.41B
	Mild-TID	302.67fg ± 0.57	294.00h ± 0.54	309.33f ± 2.44	297.67gh ± 1.81	283.33i ± 0.83	319.00e ± 0.27		
	Sever-TID	184.33n ± 1.23	193.67m ± 1.66	205.33l ± 0.83	191.67m ± 1.97	238.67k ± 0.42	247.67j ± 0.68		
Year-II	Normal	334.67cd ± 1.23	337.33c ± 1.91	328.33de ± 0.96	297.33gh ± 1.81	208.33k ± 0.68	206.33kl ± 0.96		291.98A
	Mild-TID	290.33h ± 1.85	355.67b ± 1.13	244.33j ± 1.23	324.00ef ± 1.03	200.00l ± 1.09	268.67i ± 1.77		
	Sever-TID	323.67ef ± 1.77	383.67a ± 1.44	305.33g ± 0.16	329.33c. e ± 1.29	199.33l ± 1.40	319.00f ± 1.52		
		LSD*p*0.05 {Seed priming, Seed rate, TID} Year-I ± 7.1122, Year-II ± 8.2943, Interaction 1.8090							
Number of grains spike^−1^									
Year-I	Normal	37.60g ± 0.60	40.70e ± 0.53	40.83e ± 0.51	45.90b ± 0.57	36.76h ± 0.47	49.93a ± 0.59		39.83B
	Mild-TID	36.80h ± 0.53	40.66e ± 0.58	38.70f ± 0.60	41.90d ± 0.63	39.03f ± 0.56	41.53d ± 0.60		
	Sever-TID	33.90k ± 0.65	42.60c ± 0.62	35.60i ± 0.61	38.00g ± 0.54	34.88j ± 0.53	41.71d ± 0.48		
Year-II	Normal	42.66fg ± 0.66	50.76b ± 0.64	46.83cd ± 0.60	46.90cd ± 1.31	42.73fg ± 0.65	54.63a ± 0.22		45.18A
	Mild-TID	45.70de ± 0.64	46.70cd ± 0.64	42.03gh ± 0.67	45.66de ± 0.67	41.93gh ± 0.75	49.86b ± 0.65		
	Sever-TID	40.70hi ± 0.65	43.90ef ± 0.63	36.73j ± 0.59	48.00c ± 0.64	39.66i ± 0.67	47.96c ± 0.65		
		LSD*p*0.05 {Seed priming, Seed rate, TID} Year-I ± 0.4090, Year-II ± 1.8419, Interaction 0.3249							
1000 Grains weight (g)									
Year-I	Normal	38.22cd ± 0.20	43.21b ± 0.08	36.98cd ± 0.15	43.34b ± 0.22	41.41b ± 0.30	52.94a ± 0.49		38.31
	Mild-TID	32.38e ± 0.20	33.45e ± 0.24	37.02cd ± 0.49	37.86cd ± 0.35	36.42d ± 0.66	38.96c ± 0.77		
	Sever-TID	29.07f ± 0.35	33.40e ± 0.64	33.47e ± 0.35	38.50cd ± 0.27	41.80b ± 0.49	41.21b ± 0.81		
Year-II	Normal	31.98kl ± 0.47	37.59gh ± 0.33	39.52ef ± 0.19	47.06b ± 0.52	41.63c ± 0.27	54.01a ± 0.40		38.03
	Mild-TID	33.83j ± 0.34	30.53mn ± 0.23	39.77df ± 0.15	41.04cd ± 0.12	38.73fg ± 0.15	40.33c.e ± 0.21		
	Sever-TID	33.26jk ± 0.40	32.81jk ± 0.38	30.65lm ± 0.39	36.59hi ± 0.38	29.29n ± 0.25	35.40i ± 0.25		
		LSD*p*0.05 {Seed priming, Seed rate, TID} Year-I ± 2.1830, Year-II ± 1.3504, Interaction Non-significant							
Grains yield (t ha^−1^)									
Year-I	Normal	3.47g ± 0.16	3.53g ± 0.25	4.07d ± 0.21	4.93b ± 0.19	3.54g ± 0.18	5.69a ± 0.26		3.64A
	Mild-TID	3.45g ± 0.19	3.78f ± 0.23	3.79f ± 0.24	3.98de ± 0.14	3.42g ± 0.22	4.30c ± 0.24		
	Sever-TID	2.24j ± 0.10	3.05h ± 0.18	2.67i ± 0.13	2.66i ± 0.05	3.08h ± 0.16	3.88ef ± 0.12		
Year-II	Normal	3.46e ± 0.33	3.64ce ± 0.11	3.81bd ± 0.27	4.08b ± 0.57	3.83bd ± 0.24	5.88a ± 0.07		3.18B
	Mild-TID	3.50de ± 0.36	2.06h ± 0.16	2.63fg ± 0.31	2.93f ± 0.24	2.93f ± 0.35	4.07b ± 0.36		
	Sever-TID	1.89 h ± 0.15	2.01h ± 0.22	1.42i ± 0.14	2.46g ± 0.17	2.76fg ± 0.28	3.97bc ± 0.34		
		LSD*p*0.05 {Seed priming, Seed rate, TID} Year-I ± 0.1483, Year-II ± 0.3463, Interaction 0.0629							
Biological yield (t ha^−1^)									
Year-I	Normal	12.42e ± 0.40	12.58e ± 0.62	13.92c ± 0.53	16.08b ± 0.47	12.60e ± 0.44	17.97a ± 0.66		11.39B
	Mild-TID	11.14g ± 0.48	11.95f ± 0.57	11.97f ± 0.59	12.46e ± 0.34	11.06g ± 0.56	13.25d ± 0.61		
	Sever-TID	6.24k ± 0.24	8.25i ± 0.44	7.30j ± 0.34	7.27j ± 0.12	8.32i ± 0.39	10.33h ± 0.30		
Year-II	Normal	14.29ef ± 0.82	14.72de ± 0.27	15.16cd ± 0.67	15.82bc ± 1.43	15.19cd ± 0.61	20.96a ± 0.17		13.30A
	Mild-TID	15.01ce ± 0.89	11.40h ± 0.39	12.84g ± 0.77	13.57fg ± 0.60	13.59fg ± 0.86	16.43b ± 0.91		
	Sever-TID	8.47j ± 0.38	8.79j ± 0.55	7.32k ± 0.35	9.90i ± 0.42	10.66hi ± 0.70	15.31cd ± 0.84		
		LSD*p*0.05 {Seed priming, Seed rate, TID} Year-I ± 0.3724, Year-II ± 0.8687, Interaction 0.1579							
Harvest index (%)									
Year-I	Normal	41.55	45.11	42.02	41.86	38.44	44.08		39.53A
	Mild-TID	34.17	39.05	36.54	35.52	34. 80	39.78		
	Sever-TID	34.79	41.09	39.29	40.61	41.92	41.00		
Year-II	Normal	41.93	39.89	33.17	41.00	40.62	37.84		38.38B
	Mild-TID	37.11	38.08	34.86	37.46	39.75	41.22		
	Sever-TID	34.69	41.53	35.81	39.62	37.71	38.58		
		LSD*p*0.05 {Seed priming, Seed rate, TID} Non-significant, Interaction 0.9945							

Different letters represent the level of significant among treatment means at probability level @ 5%.

### Plant anti-oxidant defense behavior

The interactive effect of seed priming, seed rate, under terminal irrigation drought (TID) on the total soluble protein was significantly shown in Table 4. It was observed that plants planted in the treatment applied MLE30-priming technique with curtailed seed rate (25 kg ha^−1^) obtained maximum production of total soluble protein under induced severe-TID followed by mild-TID as compared to normal conditions during the year-II of the trial (Table 4). The values of enzymatic contents including superoxide dismutase, peroxidase, catalase in wheat plants having MLE30-priming using curtailed seed rate in severe-TID condition were noted highest, average values in mild-TID and lowest in control during the 1st year of trial as per 2nd year presented in the Table 4. In the same way, MLE30-priming augmented the non-enzymatic activities; ascorbic acid, total phenolic contents observed maximum in the wheat plants leaves having curtailed seed rate as compared to recommended seed rate under severe-TID condition during the year-II as per year-I (Table 4).

**Table 4:** Evaluate the organic seed priming with curtailed seed rate on physiological anti-oxidant contents of wheat crop under terminal irrigation drought (TID).

Seed priming		Control		Hydro-priming		MLE30-priming		Mean
Seed rate/TID		Recommended 125 kg ha^−1^	Curtailed 25 kg ha^−1^	Recommended 125 kg ha^−1^	Curtailed 25 kg ha^−1^	Recommended 125 kg ha^−1^	Curtailed 25 kg ha^−1^	
Total soluble protein (mg g^−1^)								
Year-I	Normal	0.60g ± 0.11	0.82f ± 0.24	0.84f ± 0.18	1.43bd ± 0.17	1.40cd ± 0.15	1.49bc ± 0.26	1.31B
	Mild-TID	1.09e ± 0.09	1.12e ± 0.16	1. 25de ± 0.33	1.53bc ± 0.26	1.60bc ± 0.23	1. 56bc ± 0.27	
	Sever-TID	1.21de ± 0.17	1.25de ± 0.25	1.16e ± 0.15	1.62bc ± 0.27	1.63b ± 0.32	1.93a ± 0.10	
Year-II	Normal	1.97ef ± 0.09	1.94fg ± 0.14	1.85ij ± 0.02	1.79jk ± 0.05	1.70l ± 0.09	1.87hi ± 0.03	1.95A
	Mild-TID	1.88gi ± 0.26	1.77k ± 0.60	2.03ce ± 0.30	1.99df ± 0.36	1.83ik ± 0.41	2.05bc ± 0.44	
	Sever-TID	1.93fh ± 0.12	2.01ce ± 0.16	1.97ef ± 0.19	2.12b ± 0.14	2.05cd ± 0.12	2.30a ± 0.92	
		LSD*p*0.05 {Seed priming, Seed rate, TID} Year-I ± 0.2223, Year-II ± 0.0660, Interaction 0.0565						
Superoxide dismutase (IU min^−1^ mg^−1^ protein)								
Year-I	Normal	55.50f ± 1.09	75.59d ± 1.32	62.65e ± 0.94	63.14e ± 0.49	64.43e ± 1.33	67.33e ± 0.89	70.93B
	Mild-TID	63.90e ± 0.57	74.53d ± 1.21	64.52e ± 0.62	73.81d ± 0.67	46.65g ± 1.77	74.70d ± 0.42	
	Sever-TID	94.50c ± 0.89	104.69b ± 0.75	47.46g ± 1.71	66.45e ± 0.43	100.10bc ± 1.58	125.94a ± 0.83	
Year-II	Normal	58.21i ± 0.41	67.70h ± 1.15	73.65g ± 0.94	74.14g ± 0.49	66.50h ± 1.09	86.59f ± 1.32	88.09A
	Mild-TID	62.88hi ± 0.32	107.32b ± 0.32	50.64j ± 0.76	94.22de ± 0.24	67.46h ± 1.71	86.45f ± 0.43	
	Sever-TID	88.97ef ± 0.62	98.26cd ± 0.67	101.60c ± 0.89	111.79b ± 0.75	107.20b ± 1.58	133.04a ± 0.83	
		LSD*p*0.05 {Seed priming, Seed rate, TID} Year-I ± 6.2420, Year-II ± 5.2595, Interaction 1.3162						
Peroxidase (mmol min^−1^ mg protein^−1^)								
Year-I	Normal	20.32j ± 0.29	22.73fg ± 0.15	21.96gi ± 0.17	23.68ef ± 0.09	20.95ij ± 1.91	24.50de ± 0.41	23.68A
	Mild-TID	21.65hi ± 0.14	20.38j ± 0.17	24.39e ± 0.10	25.69c ± 0.13	22.78fg ± 0.11	27.80b ± 0.28	
	Sever-TID	19.27k ± 0.22	22.62gh ± 0.42	23.74ef ± 0.08	26.48c ± 0.24	25.49cd ± 0.13	31.79a ± 0.09	
Year-II	Normal	19.62i ± 0.18	19.02j ± 0.27	22.79ef ± 0.17	23.07e ± 0.12	22.32f ± 0.24	24.25d ± 0.14	22.43B
	Mild-TID	21.69g ± 0.14	20.42h ± 0.17	24.43cd ± 0.10	25.73b ± 0.13	22.82e ± 0.11	27.84a ± 0.28	
	Sever-TID	18.28k ± 0.11	20.11h ± 0.28	21.61g ± 0.20	23.18e ± 0.42	21.78g ± 0.44	24.79c ± 0.30	
		LSD*p*0.05 {Seed priming, Seed rate, TID} Year-I ± 1.0336, Year-II ± 0.4708, Interaction 0.1838						
Catalase (μmol min^−1^ mg protein^−1^)								
Year-I	Normal	16.90l ± 0.28	20.35h ± 0.20	14.67m ± 0.11	14.83m ± 0.12	19.38i ± 0.10	18.78j ± 0.15	21.99B
	Mild-TID	18.82j ± 0.21	18.21k ± 0.31	22.21f ± 0.14	22.23f ± 0.16	21.77g ± 0.29	23.85e ± 0.09	
	Sever-TID	21.74g ± 0.12	24.09e ± 0.13	26.82d ± 0.13	29.89b ± 0.28	27.38c ± 0.15	33.91a ± 0.11	
Year-II	Normal	12.69o ± 0.01	15.17n ± 0.17	22.14j ± 0.10	21.45k ± 0.16	19.50l ± 0.31	23.14i ± 0.21	23.63A
	Mild-TID	15.14n ± 0.20	15.63m ± 0.12	25.94f ± 0.20	25.91f ± 0.20	25.04g ± 0.31	27.05e ± 0.55	
	Sever-TID	21.44k ± 0.12	23.77h ± 0.13	29.84d ± 0.14	33.23b ± 0.32	30.63c ± 0.17	37.67a ± 0.12	
		LSD*p*0.05 {Seed priming, Seed rate, TID} Year-I ± 0.3118, Year-II ± 0.3882, Interaction 0.0809						
Ascorbic acid (m mole g^−1^)								
Year-I	Normal	57.32i ± 0.48	58.46i ± 0.44	66.59gh ± 0.45	71.99ef ± 0.41	68.10g ± 0.47	74.80cd ± 0.58	71.15B
	Mild-TID	53.69j ± 0.51	71.68f ± 0.95	71.15f ± 0.27	76.38bd ± 0.47	74.39de ± 0.42	76.21bd ± 0.50	
	Sever-TID	64.70h ± 0.53	65.86gh ± 0.55	77.05bd ± 0.49	77.08bc ± 0.32	77.51b ± 0.21	97.71a ± 0.82	
Year-II	Normal	62.97hi ± 1.44	65.44 h ± 1.24	74.64de ± 1.43	75.02ce ± 1.53	73.34ef ± 1.56	78.34b ± 1.32	72.38A
	Mild-TID	61.15i ± 0.50	70.29 fg ± 0.53	51.72j ± 0.49	76.11be ± 0.49	77.24bd ± 0.57	78.32b ± 0.40	
	Sever-TID	69.67 g ± 0.48	74.92de ± 0.52	75.97be ± 1.14	78.06bc ± 0.54	77.73bd ± 0.54	81.90a ± 0.76	
		LSD*p*0.05{Seed priming, Seed rate, TID} Year-I ± 2.6754, Year-II ± 3.0983, Interaction 0.6616						
Total phenolic contents (mg g^−1^)								
Year-I	Normal	0.80gh ± 0.15	0.64gh ± 0.05	0.65gh ± 0.06	0.36h ± 0.09	1.14fg ± 0.45	1.54ef ± 0.51	1.64B
	Mild-TID	0.84gh ± 0.17	1.16fg ± 0.04	1.14fg ± 0.25	1.24fg ± 0.41	1.12fg ± 0.55	1. 93de ± 0.12	
	Sever-TID	2.05ce ± 0.80	2.60bc ± 0.23	2.92b ± 0.07	3.06b ± 0.06	2.57b.d ± 0.03	3.77a ± 0.22	
Year-II	Normal	1.06l ± 0.02	2.16j ± 0.41	1.88jk ± 0.43	2.74i ± 0.34	1.81k ± 0.64	2.91i ± 0.47	3.15A
	Mild-TID	2.98hi ± 0.14	3.32fg ± 0.34	3.44eg ± 0.44	3.79cd ± 0.48	3.30gh ± 0.46	4.07bc ± 0.44	
	Sever-TID	3.65de ± 0.24	3.62dg ± 0.22	3.63df ± 0.49	3.37eg ± 0.51	4.26b ± 0.12	4.82a ± 0.05	
		LSD*p*0.05 {Seed priming, Seed rate, TID} Year-I ± 0.6659, Year-II ± 0.3265, Interaction 0.1198						

Different letters represent the level of significant among treatment means at probability level @ 5%.

### Chlorophyll contents, K^+^ contents, and water use efficiency

**Figure 2:**
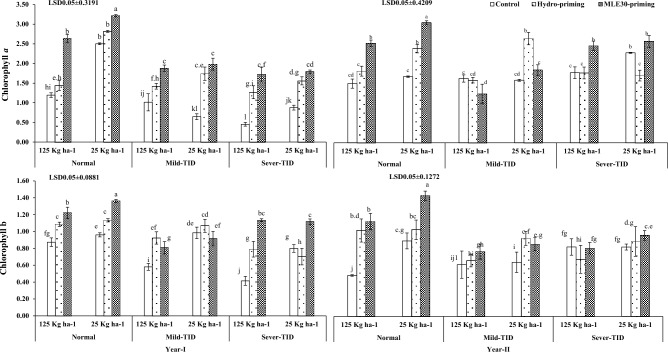
Effect of seed priming with curtailed seed rate on Chlorophyll *a* & *b* (mg g^−1^) of wheat under terminal irrigation drought (TID).

**Figure 3:**
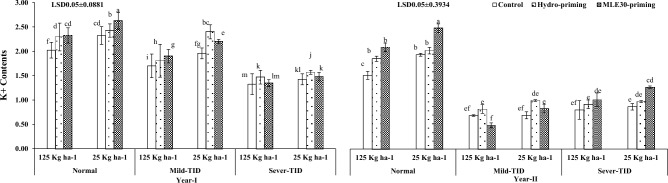
Effect of seed priming with curtailed seed rate on K^+^ (mg g^−1^) contents of wheat under terminal irrigation drought (TID).

**Figure 4:**
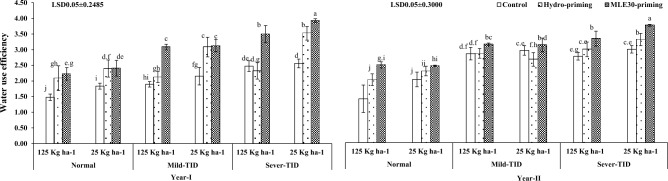
Effect of seed priming with curtailed seed rate on water use efficiency of wheat under terminal irrigation drought (TID).

Figure 2 illustrated the positive effect of MLE30-priming in generating the maximum chlorophyll contents “*a*” and “*b*” followed by hydro-priming, and control treatments with curtailed seed rate as per recommended under severe-TID and mild-TID after normal irrigation conditions during both years of trials. The observation depicted the significant results related to leaf K^+^ contents in wheat crop treated with the MLE30-priming technique and maximum K^+^ contents were obtained by applying the curtailed seed rate in normal irrigation conditions as compared to severe-TID during year-I as per year-II shown in the Fig. 3. On the contrary, the MLE30-priming technique enhanced the plant water use efficiency with curtailed seed rate under severe-TID and mild-TID as per normal irrigation conditions during both years of exploration (Fig. 4).

### Economic analysis

Table 5 indicated the average economic analysis that wheat crop sown treated with MLE30-priming in normal irrigation conditions revealed the highest net income and benefit–cost ratio (BCR) as per induced mild and severe-TID. The applied curtailed seed rate treatment achieved the greatest BCR values and net economic returns as compared to the recommended seed rate.

**Table 5:** Average economic analysis of wheat crop during the years 2021–2022 and 2022–2023.

Seed priming	Terminal irrigation drought (TID)	Seed rate	Total expenditure (US$ ha^−1^)	Gross income (US$ ha^−1^)	Net income (US$ ha^−1^)	Benefit cost ratio
Control	Normal	Recommended 125 kg ha^−1^	330.34	681.71	351.37	1.06
		Curtailed 25 kg ha^−1^	323.02	709.44	386.42	1.20
	Mild-TID	Recommended 125 kg ha^−1^	344.98	772.70	427.72	1.24
		Curtailed 25 kg ha^−1^	308.39	484.60	176.21	0.57
	Sever-TID	Recommended 125 kg ha^−1^	313.27	437.45	124.18	0.40
		Curtailed 25 kg ha^−1^	300.83	401.47	100.64	0.33
Hydro-priming	Normal	Recommended 125 kg ha^−1^	349.85	701.81	351.96	1.01
		Curtailed 25 kg ha^−1^	327.90	687.10	359.20	1.10
	Mild-TID	Recommended 125 kg ha^−1^	335.22	589.31	254.09	0.76
		Curtailed 25 kg ha^−1^	313.27	628.72	315.46	1.01
	Sever-TID	Recommended 125 kg ha^−1^	308.39	321.89	13.50	0.04
		Curtailed 25 kg ha^−1^	295.95	511.72	215.77	0.73
MLE30-priming	Normal	Recommended 125 kg ha^−1^	354.73	743.13	388.40	1.09
		Curtailed 25 kg ha^−1^	332.78	1007.48	674.70	2.03
	Mild-TID	Recommended 125 kg ha^−1^	340.10	670.87	330.77	0.97
		Curtailed 25 kg ha^−1^	318.15	814.61	496.46	1.56
	Sever-TID	Recommended 125 kg ha^−1^	313.27	536.70	223.43	0.71
		Curtailed 25 kg ha^−1^	300.83	817.97	517.14	1.72

### Biplot and principal component analysis (PCA)

**Figure 5:**
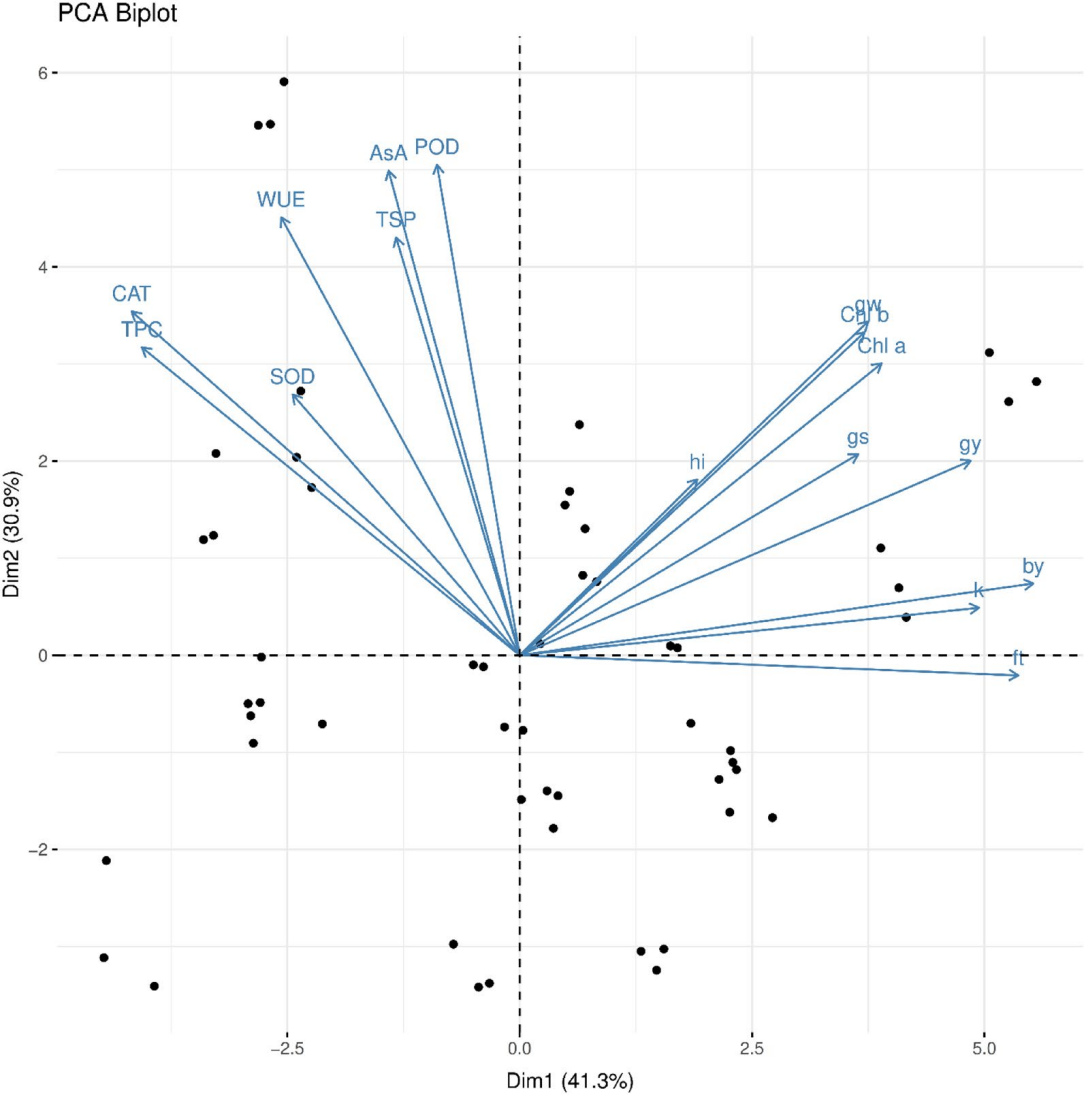
Biplot analysis for principal component analysis (PCA) of growth, yield, and biochemical attributes of wheat crop. Productive/fertile tillers (ft), Number of grains spike^−1^ (gs), 1000 Grains weight (gw), Grain’s yield (gy), Biological yield (by), Harvest index (hi), Total soluble protein (TSP), Superoxide dismutase (SOD), Peroxidase (POD), Catalase (CAT), Ascorbic acid (AsA), Total phenolic contents (TPC), Chlorophyll *a* & *b* (Chl a, b), K^+^ contents (k), Water use efficiency (WUE).

Figure 5 illustrated the positive interaction among the growth, yield, biochemical attributes under the applied treatments of organic seed priming, terminal drought, curtailed seed rate in wheat crop during both years of the project study. The observed parameters depicted a significantly similar trend of results after the application of treatments under biplot analysis (Fig. 5).

## Discussion

Observations proved that seeds obtained after terminal irrigation drought (TID) stress in wheat plants illustrated the improving performance via tolerance against the applied drought conditions. The plants treated with the applied terminal drought, especially during the crop reproductive growth stages received the behavioral modifications by inducing drought resistance as per normal irrigation conditions^2^. It has been noted that drought stress impacted the seed composition and minimized the availability of moisture contents, diminished the ash formations, and reduced the crude fate development while, improving the anti-oxidant defense system, total soluble proteins, and total phenolic contents under the normal treatment of irrigation application^10^. Plants altered the relations between the source-sink for better translocating and accumulating the sugar contents during the anthesis and grain filling stages under the induced terminal irrigation drought stress condition^7^. Various scientists presented the research findings in cereal crops that the applied terminal drought stress impaired the starch & sucrose production and augmented the soluble & heat shock proteins for improving the seed protein contents^9,11,30^. Wheat plants were observed in ameliorating the synthesis of grain protein contents under the applied drought stress at post-anthesis stages. The anti-oxidants defense system responded positively against reactive oxygen species (ROS) translocated into the seeds for stress tolerance under unfavorable environmental conditions of terminal irrigation drought^10,12^.

Two years of this project study illustrated that the reduction in yield and yield-related attributes observed by quenching the water relation might be the reason for creating the oxidative stress in the plants during the severe and mild TID applied at reproductive growth stages (flowering and milking) and (flowering) respectively. However, the applied MLE30-priming may be the reason to enhance the enzymatic (SOD, POD, CAT) and non-enzymatic (AsA, TPC) contents for compensating the drastic effect and favored the wheat grains production under TID stress condition^31^. The improving trend in the number of fertile tillers, number of grains spike^−1^, and 1000-grains weight under mild-TID followed by severe-TID might be due to treatment applied MLE30-priming with curtailed seed rate at 25 kg ha^−1^ which provided the wheat plants to germinate and grow with sufficient availability of input resources including’s nutrients, space, light, and moisture for performing the complete phenological stages^14^. Wheat grains product is the outcome of crop received better under the applied treatment of organic MLE30-priming with curtailed seed rate @ 25 kg ha^−1^ in mild-TID followed by severe-TID after the normal irrigation condition might be the reason for generating the tolerance by active anti-oxidant defense system behavior.

Wheat crop faced a sensitive phase under the applied terminal irrigation drought including severe-TID and mild-TID resulted in damaging effects at the cellular oxidative level. Reactive oxygen species (ROS) quenched the plant molecular oxygen and reduced the anti-oxidants production leading to a serious threat to plant survival^32^. The prominent activity of anti-oxidant defense system (enzymatic and non-enzymatic contents) diminished the toxic-free ROS productions and boosted the plant tolerance to face the harsh conditions of terminal irrigation drought. The purpose of this project study is to ameliorate the anti-oxidants against the injurious ROS activities by the applied treatment of organic MLE30-priming approach while providing vigorous germination potential and suitable growing environment under curtailed seed rate at 25 kg ha^−1^ during the severe-TID and mild-TID conditions after normal irrigation conditions^17^. Observations illustrated that the action of SOD detoxified the superoxide anion radical (O^2·−^) into oxygen compound (O_2_), CAT converted hydrogen peroxide (H_2_O_2_) into hydrogen oxide (H_2_O) and oxygen molecules (O_2_). Moreover, treated wheat seeds with organic MLE30-priming plus curtailed seed rate stimulated the POD production against H_2_O_2_ activities, AsA mitigated the excessive generation of HO_2_ and 1O^2^ during the applied terminal irrigation drought stress^16^. Organic MLE30-priming motivated the plants to release healthy TPC values against ROS by facilitating photosynthetic activities under severe-TID followed by mild-TID as per normal irrigation conditions. Wheat crop received tolerance after the applied treatment of MLE30-priming along with curtailed seed rate might be the reason to boost up anti-oxidant defense system for alleviating the ROS activities during both terminal irrigation drought conditions. The treated plants with organic MLE30-priming enhanced the favorable condition by making homeostasis condition between the anti-oxidant & ROS contents at the cellular level^11^. So, it is evidenced that applying organic MLE30-priming with a curtailed seed rate may ameliorate the enzymatic and non-enzymatic anti-oxidant defense system and resulted in producing wheat grains production during the terminal irrigation drought.

Results illustrated the significant impact of curtailed seed rate @ 25 kg ha^−1^ in producing the higher grains yield due to optimum rise in fertile/productive tillers might be the reason for less competition for plant-plant, light, water, and shade during the wheat production^32^. The application of a curtailed seed rate provided better space to develop the plant tillers and showed fewer evaporation losses during the terminal irrigation drought stress conditions. It has been observed that the utilization of optimum spacing with curtailed seed rate improved grain production by healthy off-setting formation during unfavorable environmental drought stress conditions^18^. On the other hand, the most important point is noted that the increasing trend of grains production under applied treatment of organic MLE30-priming with curtailed seed rate compensated the yield losses and boosted the development of productive tillers, grains spike-1, 1000-grains weight, and biological yield might be due to the presence of optimum availability of input resources during the crop husbandry condition^12^.

Organic MLE30-priming proved the establishment of the early, vigorous & synchronized seedling in wheat plants might be the reason for fulfilling the basic requirements efficiently during the crop phenological growth stages under the induced severe-TID followed by mild-TID conditions. Due to the effective results of productive seedlings, MLE30 seed priming enhanced the chlorophyll contents, water use efficiency (WUE), and potassium (K^+^) contents leading to obtaining healthy grains production might be the reason for the maximum availability of dry matter assimilations and photosynthates during the grains filling and development stages^25,27^. MLE30-priming along with curtailed seed rate triggered the potassium (K^+^) contents especially during the reproductive wheat growth stages, helpful as a catalyst to perform the various biochemical, physiological, and morphological processes^33^.

Recently, progressive farmers believed in adopting advanced approaches by considering the most marketable demand and commercial feasibility in terms of cost-effectiveness and input charges^12^. The supremacy feedback of the applied treatments MLE30-priming with curtailed seed rate achieved in term of higher net income and BCR might be due to the cost-effective preparation and application during the field trial economic analysis for wheat production^34^.

## Conclusion

The present project study strengthens the diversified concept of organic MLE30-priming to support in obtaining the maximum grains yield by triggering the anti-oxidant defense system in wheat crop under terminal irrigation drought. Moreover, the combine use of organic MLE30-priming with curtailed seed rate (25 kg ha^−1^) is the best agronomic and sustainable approach for saving the seed losses during the crop husbandry condition for coping the water stress condition during the wheat crop phenological growth stages. Also, MLE30-priming will be upgraded on the basis of nutritional profiling by the use proteinaceous banding profiling (SDS-page) for getting the biofortified wheat grains production in the future prospective.

## Data Availability

All data generated or analyzed during this study are included in this published article.

## Abbreviations

MLE: Moringa leaf extract
TID: Terminal irrigation drought
TSP: Total soluble protein
SOD: Superoxide dismutase
POX: Peroxidase
CAT: Catalase
AsA: Ascorbic acid
TPC: Total phenolic content
ROS: Reactive oxygen species
RCBD: Randomized complete block design
T: Tillering
B: Booting
F: Flowering
M: Milking
K^+^: Potassium
WUE: Water use efficiency
BCR: Benefit cost ratio
LSD: The least significant difference
HI: Harvest index
O^2·-^: Superoxide anion radical
O_2_: Oxygen compound
H_2_O_2_: Hydrogen peroxide
H_2_O: Hydrogen oxide
HO_2_: Hydroperoxyl
1O^2^: Singlet molecular oxygen

## References

[1] Nawaz H, Hussain N, Ahmad N, Rehman H, Alam J (2021). Efficiency of seed bio-priming technique for healthy mungbean productivity under terminal drought stress. J. Integr. Agric..

[2] Nawaz A, Farooq M, Cheema SA, Yasmeen A, Wahid A (2013). Stay green character at grain filling ensures resistance against terminal drought in wheat. Int. J. Agric. Biol..

[3] Lafitte HR, Yongsheng G, Yan S, Li ZK (2007). Whole plant responses, key processes, and adaptation to drought stress: The case of rice. J. Exp. Bot..

[4] Nawaz H, Hussain N, Anjum MA, Rehman H, Jamil M, Raza MAS, Farooq O (2019). Biochar amendment with irrigation water-regimes at tillering and booting stages enhanced physiological and antioxidant behaviour for wheat productivity. Int. J. Agric. Biol..

[5] Leport L, Turner NC, French RJ, Barr MD, Duda R, Davies SL (2006). Physiological responses of chickpea genotypes to terminal drought in a Mediterranean-type environment. Eur. J. Agron..

[6] Farooq M, Basra SMA, Wahid A (2006). Priming of field-sown rice seed enhances germination, seedling establishment, allometry and yield. Plant Growth Regul..

[7] Royo C, Abaza M, Blanco R, Moral LFG (2000). Triticale grain growth and morphometry as affected by drought stress, late sowing, and simulated drought stress. Aust. J. Plant Physiol..

[8] Jafar MZ, Farooq M, Cheema MA, Afzal I, Basra SMA, Wahid MA, Aziz T, Shahid M (2012). Improving the performance of wheat by seed priming under saline conditions. J. Agron. Crop Sci..

[9] Karim MA, Hamid A, Rahman S (2000). Grain growth and yield performance of wheat under subtropical conditions: II. Effect of water stress at reproductive stress. Cereal Res. Commun..

[10] Reddy AR, Chaitanya KV, Vivekanandan M (2004). Drought-induced responses of photosynthesis and antioxidant metabolism in higher plants. J. Plant Physiol..

[11] Farooq M, Wahid A, Kobayashi N, Fujita D, Basra SMA (2009). Plant drought stress: Effects, mechanisms, and management. Agron. Sustain. Dev..

[12] Nawaz H, Hussain N, Yasmeen A, Bukhari SAH, Hussain MB (2017). Seed priming: A potential stratagem for ameliorating soil water deficit in wheat. Pak. J. Agric. Sci..

[13] Apel K, Hirt H (2004). Reactive oxygen species: Metabolism, oxidative stress, and signal transduction. Annu. Rev. Plant Biol..

[14] Ali Z, Basra SMA, Munir H, Mahmood A, Yousaf S (2011). Mitigation of drought stress in maize by natural and synthetic growth promoters. J. Agric. Soc. Sci..

[15] Kaya MD, Okçub G, Ataka M, Çıkılıc Y, Kolsarıcıa Ö (2006). Seed treatments to overcome salt and drought stress during germination in sunflower (*Helianthus annuus* L.). Eur. J. Agron..

[16] Nawaz H, Yasmeen A, Anjum MA, Hussain N (2016). Exogenous application of growth enhancers mitigate water stress in wheat by antioxidant elevation. Front. Plant Sci..

[17] Yasmeen A, Basra SMA, Farooq M, Rehman H, Hussain N, Athar HR (2013). Exogenous application of moringa leaf extract modulates the antioxidant enzyme system to improve wheat performance under saline conditions. Plant Growth Regul..

[18] Rajala A, Niskanen M, Isolahti M, Peltonen-Sainio P (2011). Seed quality effects on seedling emergence, plant stand establishment and grain yield in two-row barley. Agr. Food Sci..

[19] Amanullah KA, Zahid H, Jan D (2010). Performance of wheat cultivars sown at different seeding rates under drought-stress conditions. Arch. Agron. Soil Sci..

[20] Bradford M (1976). A rapid and sensitive method for the quantitation of microgram quantities of protein utilizing the principle of protein-dye binding. Ann. Biochem..

[21] Chance M, Maehly AC (1954). The assay of catalases and peroxidases. Methods Biochem. Anal..

[22] Giannopolitis CN, Reis SK (1997). Superoxide dismutase I. Occurrence in higher plants. Plant Physiol..

[23] Ainsworth EA, Gillespie KM (2007). Estimation of total phenolic content and other oxidation substrates plant in tissues using Folin-Ciocalteu reagent. Nat. Prot..

[24] Waterhouse AL, Wrolstad RE (2001). Determination of total phenolics. Current Protocols in Food Analytical Chemistry.

[25] Nagata M, Yamashita I (1992). Simple method for simultaneous determination of chlorophyll and carotenoids in tomato fruit. J. Jpn. Soc. Food Sci. Technol..

[26] Rashid, A. *Mapping Zinc Fertility of Soils Using Indicator Plants and Soils-Analyses*. Ph.D. Dissertation, University of Hawaii, 1–379 (1986).

[27] Viets FG (1962). Fertilizers and the efficient use of water. Adv. Agron..

[28] Dewey DR, Lu KH (1959). A correlation and path coefficient analysis of components of crested wheat grass and seed production. J. Agron..

[29] Steel RGD, Torrie JH, Dicky DA (1997). Principles and Procedures of Statistics, A Biometrical Approach.

[30] Sairam RK, Srivastava GC, Agarwal S, Meena RC (2005). Differences in antioxidant activity in response to salinity stress in tolerant and susceptible wheat genotypes. Biol. Plant..

[31] Yasmeen A, Basra SMA, Ahmad R, Wahid A (2012). Performance of late sown wheat in response to foliar application of *Moringa oleifera* Lam. Leaf extract. Chil. J. Agric. Res..

[32] Ullah A, Farooq M, Nadeem F, Rehman A, Nawaz A, Naveed M, Wakeel A, Hussain M (2020). Zinc seed treatments improve productivity, quality, and grain biofortification of desi and kabuli chickpea (*Cicer arietinum*). Crop Pasture Sci..

[33] Cakmak I (2005). The role of potassium in alleviating detrimental effects of abiotic stresses in plants. J. Plant Nutr. Soil Sci..

[34] Farooq S, Shahid M, Khan MB, Hussain M, Farooq M (2014). Improving the productivity of bread wheat by good management practices under terminal drought. J. Agron. Crop Sci..

